# Work and Food

**Published:** 1890-12-06

**Authors:** Andrew Clark


### Work and Food.

By Sir Andrew Clark, Bart., M.D., F.R.S.

The distaste and dislike which is growing up around us with regard to work is a most grievous and terrible mistake, for the highest life that any man or woman can reach in this world is associated with the fullest work. If those who know the value of life were to be asked which are the most worthy—those who work much too much or those who work much too little, they would at once answer that those who work much too much are worth twenty times those who work much too little. Let mothers who have daughters whom they do not know what to do with, and who are becoming plagues to themselves and to you, fill their lives with work, and they will then have no need to complain of them.

There is growing up in the nursing world a habit of having far too frequent meals in the day, and far too much variety at them. Meals should not be multiplied in the way they now are. The plan of having tea before breakfast, lunch at eleven o'clock, then dinner, tea again in the afternoon, and supper at night is about the worst plan that can be adopted. Nurses should have four meals a-day, four good, sensible meals of fresh, simple, nourishing food. Women have but one notion about food, and that is that the oftener they eat and the more varied the food is the better they will be. This is a mistake. Four simple meals, meals of plain, nourishing food, and nothing between them, is the best way of sustaining the health and of supporting the body. Above all tinned meats should be avoided, as they form a most dangerous kind of food. The diet of twenty-five years ago, when nurses had plain bread and butter for breakfast at half-past six, a dinner consisting of a joint at one, and a cold meat supper, was much more fitting for the maintenance of health and the support of the working powers than the diet of the present day.

